# Diseases of the Heart and Circulation

**Published:** 1903-04-11

**Authors:** 


### Diseases of the Heart and Circulation.

**Cyanosis.**—Some fresh observations are given by Gibson[1] in support of some theories put forward by him a few years ago. The one constant factor in the production of cyanosis is diminution of oxygenation, which may be the result of respiratory affections which hinder the access of air or diminish the area of the aerating surface, or may be due to circulatory disorders lessening the amount of blood flowing through the lungs. The blood in cyanosis is of high specific gravity—from 1,070 to 1,080, the amount of hæmoglobin is large, whilst the number of erythrocytes increases so that it frequently exceeds 7,500,000 per cubic millimetre, and the leucocytes often reach such a figure as 12,000 per cubic millimetre. The explanation of these facts, previously advanced by Gibson, is that they are due to lessened wear and tear of the corpuscles, so that the duration of their individual existence is increased, and their number is proportionately augmented until a balance is struck between their production and destruction. The lessened tear and wear is caused by the fact that they are insufficiently oxygenated and do not play such an active part as oxygen carriers, and also by the lessened metabolism of the tissues. Such a theory is now supported by the additional observations that in cyanosis there is a general increase throughout the whole circulation in the amount of hæmoglobin and number of erythrocytes, whilst the state of matters as regards the leucocytes is less constant. Inhalation of oxygen, even for long periods of time, has no effect on cyanosis, and, further, is not found to diminish the number of erythrocytes per cubic millimetre. This is due to the now generally recognised fact that the ordinary air of the atmosphere contains considerably more oxygen than can be absorbed in any circumstances.

**The Clinical Value of Blood Pressure** is the subject of papers by Briggs and Cook,[2] the former dealing with adults, the latter with children. The blood pressure was determined by a Riva-Rocci sphygmomanometer. The effects of alcohol were never marked; half to one ounce of whisky produced a temporary rise in blood pressure, never lasting over 30 minutes, and typically followed by a slight but more permanent fall below the previous level. The total effect of alcohol then on the vaso-motor system was depression. Strychnia hypodermically in moderate doses was found to produce a rise in pressure somewhat delayed as compared with that after alcohol but far more persistent—lasting from two to three hours. A similar rise, with more rapid onset and somewhat less permanent, was found to follow the administration of digitalin. The rise of blood pressure was not followed by any fall in either of these two drugs. By the routine treatment with strychnia and digitalis the blood-pressure can thus be kept constantly raised, as may be done for instance in severe cases of typhoid fever. A rational basis for the administration of alcohol must be sought in some alteration of metabolism and not in any assumed improvement in the pulse. Subcutaneous infusions of normal saline solutions have no stimulant effect on the pulse, and they are contra-indicated in surgical and traumatic shock, whose essential feature is a perilously low blood-pressure; in such cases they may actually do harm. Much the same results were found in children by Cook, the only exception being that alcohol in repeated doses appeared to have a marked and permanent beneficial effect. It is thus useful during the course of a depressing illness where, besides its doubtful stimulant effect, it is of value as a conserver of energy in the nature of a food.

**Reduplication of the First Sound of the Heart** Phear[3] has recently investigated the clinical associations of this well-known phenomenon in a considerable number of cases. The name, *bruit de galop*, first given it by Bouillaud, has the merit of being descriptively accurate, while involving no theory as to its nature. The term cantering rhythm, or *bruit de rappel*, should be reserved for instances of simulated reduplication of the second sound. Reduplication of the first sound is never perfect; it is rather an interruption in the uniformity of the sound with two points of emphasis; both parts fall within the period of ventricular systole, and they are best heard intermediate between the apex beat and the sternal margin. The common clinical conditions in which this reduplicated first sound is heard are—(1) valvular lesions, especially mitral regurgitation; (2) arterial degeneration, with raised systemic tension and accentuated aortic second sound; (3) pulmonary emphysema; (4) anæmia, chiefly of the chlorotic type; (5) in cases of dyspepsia. The second group includes cases of chronic nephritis, and is remarkable for the large number of cases in which the occupation involved contact with lead. The theory that an apparent reduplication of the first sound is really due to the interpolation of an abnormal sound immediately preceding the first sound proper, is not true, for they occur in such close proximity that they cannot occupy different phases of the cardiac cycle. Further, they do not differ in timbre, though they may in loudness. The double effect, then, is probably due to separation from one another of certain elements which, under ordinary conditions, are blended to form an undivided first sound. One factor is the vibration generated in the auriculo-ventricular valves due to the state of tension into which these membranes are abruptly thrown in the course of the ventricular systole. One part of the reduplicated first sound is attributable to mitral valve tension, the other part to tricuspid valve tension, while the general continuity of sound is due to the non-valvular components to which the unmodified first sound owes its quality of endurance. In favour of this view is the fact that the clinical associations are all such as tend to modify the pressure either in the pulmonary artery or aorta which has to be overcome before the ventricle concerned can expel its contents. It is a little remarkable that in mitral stenosis — a condition which lowers the tension in the left and raises it in the right ventricle—a reduplicated first sound is not heard. The explanation probably is that the diseased valve is so thickened that it is not capable of giving rise to a tension sound. The peculiarly sharp and abrupt quality of the first sound in this condition is probably due to the fact that it consists chiefly of an accentuated tricuspid sound and that the stiffened mitral valve has no share in its production. There is thus the same mechanism—a synchronous valve tension—in the production of reduplication both of the first and second sounds. The difference—that in the one case the reduplication is imperfect, in the other perfect—is due to the fact that the first sound is also caused by the continuous ventricular systole. Reduplication of the first sound really signifies a lengthening of the period between the closure of the valve and the production of sound on the side of the ventricle which has to meet the greater relative resistance.

**Treatment of Endocarditis.**—Huchard[4] looks on the older remedies such as the alkaline treatment, tartrated antimony, mercury, venesection, cupping, as not only useless but often harmful. Ice, though of use in pericarditis, does no good in endocarditis. The blistering treatment of Caton is also objected to, as actually harmful, and doubt is cast on the results which have been claimed for it. Salicylate of soda is the most important drug, and should be given in large doses from the very onset of any rheumatic affection and continued for a long time after the joints have recovered. Given in this way it will prevent the onset of endocarditis; it has a destructive action on the micro-organisms of rheumatism and will thus cure rheumatic endocarditis in its first or microbic phase, though it will not do so in the second or inflammatory stage which is characterised first by modifications of the tone of the heart sounds and then by murmurs. It must be remembered that endocarditis frequently comes on after joint affections have subsided. In suppurative arthritis, such as is met with in erysipelas, salicylate of soda is useless. If simple rheumatic endocarditis has occurred the best remedies are iodide of potassium and mineral waters. The former should be given in doses varying from 5 to 15 grs. daily, according to age. It tends to promote the absorption of inflammatory exudations. Mineral water treatment is more useful after an attack of acute articular rheumatism whether with or without endocarditis. Suitable hygienic and dietetic treatment is important, whilst digitalis and hydrobromate of quinine in combination improve the action of the heart if any failure occurs.

**Healing of Ulcerative Endocarditis.**—Herrick[5] has recently reviewed the evidence which shows that, exceptionally, ulcerative endocarditis may heal. The condition is fairly well recognised; there are signs of endocarditis accompanied by fever with chills, visceral embolic phenomena, petechiæ, micro-organisms in the blood, and general symptoms of a typhoid state. Such a disease is usually fatal, but cases occur in which after such a train of symptoms recovery, though with a damaged heart, takes place. Anatomical proof may be found in scarring of the valves, probably due to a destructive process having been succeeded by a reparative one. In many of the clinical cases quoted the micro-organisms found in the blood were streptococci, and the treatment was by antistreptococcic serum.

**Aneurysm of the Aorta.**—D'Espine[6] has published an account of a particular variety of aneurysm, in which a saccular diverticulum arising from the anterior surface of the aorta enters into relations with the pulmonary artery or conus arteriosus. The bulging of the aneurysmal sac has narrowed the lumen of the artery in most of the cases recorded; and in several the adhesion of the sac to the artery had excited adhesion of one or more cusps of the valve to the wall of the artery. A communication between the pulmonary artery and the aneurysm may take place. A visible pulsation in the second and third left intercostal spaces was frequently observed during life. There was a systolic bruit limited either to the third left costal cartilage, or heard all over the cardiac area with maximum intensity in the second and third left spaces. The adhesion or destruction of a valve causes a diastolic murmur of pulmonary insufficiency. Such cases present no anginal attacks, no painful phenomena, no symptoms of intrathoracic pressure; and they end like heart cases with anasarca, cachexia, and asthenia. The pressure on the pulmonary artery may cause dilatation of the right side of the heart.

[1] Lancet, Jan. 17, 1903. [2] Johns Hopkins Hospital Bulletin, Feb. 1903. [3] Lancet, Dec. 6, 1902. [4] Med. Chron., Nov. 1902 [5] Med. Chron., Sept. 1902. [6] Ibid.

